# Major ion compositions, sources and risk assessment of karst stream under the influence of anthropogenic activities, Guizhou Province, Southwest China

**DOI:** 10.7717/peerj.15368

**Published:** 2023-05-18

**Authors:** Tianhao Yang, Qixin Wu, Yanling An, Jiemei Lv

**Affiliations:** 1 School of Public Health, The Key Laboratory of Environmental Pollution Monitoring and Disease Control, Ministry of Education, Guizhou Medical University, Guiyang, China; 2 Key Laboratory of Karst Geological Resources and Environment, Ministry of Education, Guizhou University, Guiyang, China; 3 The College of Resources and Environmental Engineering, Guizhou University, Guiyang, China; 4 The College of Resources and Environmental Engineering, Guizhou Institute of Technology, Guiyang, China

**Keywords:** Major ion composition, Sources, Mining activities, Urban sewage discharge, Health risk assessment, Small karst stream

## Abstract

To explore the influence of different types of anthropogenic activity on the rivers, we investigate the major ion composition, sources and risk assessment of the karst stream (Youyu stream and Jinzhong stream), which are heavily influenced by mining activities and urban sewage, respectively. The chemical compositions of the Youyu stream water, which is heavily influenced by mining activities, are dominated by Ca^2+^ and SO_4_^2^**^–^**. However, the chemical compositions of the Jinzhong stream water, which is heavily influenced by urban sewage, are dominated by Ca^2+^ and HCO_3_**^–^**. The Ca^2+^, Mg^2+^ and HCO_3_**^–^** in Jinzhong stream are mainly derived from rock weathering, while the Youyu stream is affected by acid mine drainage, and sulfuric acid is involved in the weathering process. Ion sources analysis indicates that the Na^+^, K^+^, NO_3_**^–^**, and Cl**^–^** in the Jinzhong stream mainly derive from urban sewage discharge; but NO_3_**^–^** and Cl**^–^** of the Youyu stream mainly derive from agricultural activities, and Na^+^, K^+^ are mainly from natural sources. The element ratios analysis indicates the ratio of SO_4_^2^**^–^**/Mg^2+^ in Youyu stream (4.61) polluted by coal mine is much higher than that in Jinzhong stream (1.29), and the ratio of (Na^+^+K^+^+Cl**^–^**)/Mg^2+^ in Jinzhong stream (1.81) polluted by urban sewage is higher than Youyu stream (0.64). Moreover, the ratios of NO_3_^−^/Na^+^, NO_3_^−^/K^+^, and NO_3_^−^/Cl^−^ in the agriculturally polluted Youyu stream were higher than those in the Jinzhong stream. We can identify the impact of human activities on streams by ion ratios (SO_4_^2^**^–^**/Mg^2+^, (Na^+^+K^+^+Cl**^–^**)/Mg^2+^, NO_3_^−^/Na^+^, NO_3_^−^/K^+^, and NO_3_^−^/Cl^−^). The health risk assessment shows the HQ_T_ and HQ_N_ for children and adults are higher in Jinzhong stream than in Youyu stream and the total HQ value (HQ_T_) of children was higher than one at J1 in the Jinzhong stream, which shows that children in Jinzhong stream basin are threatened by non-carcinogenic pollutants. Each HQ value of F^−^ and NO_3_^−^ for children was higher than 0.1 in the tributaries into Aha Lake, indicating that the children may also be potentially endangered.

## Introduction

The hydrochemical characteristics of river waters could provide important insights into global and regional biogeochemical cycling of elements, and they can be used to discriminate the influence of geological factors, anthropogenic activity and atmospheric precipitation on the hydrochemistry of the watershed (Chen et al., 2002; Li et al., 2011a; Li & Zhang, 2008; Stallard & Edmond, 1983; Yang et al., 2020; Zhong et al., 2020, 2018). Recent studies have shown that human activities, such as agriculture, urbanization and exogenous acid on rock weathering. Especially in southwest China where karstification is strong, have more and more significant effects on water chemistry and water environment quality (Chen et al., 2002; Ding et al., 2017; Kou et al., 2019; Lenart-Boroń et al., 2017; Li et al., 2016a, 2011a; Meybeck, 2003; Wang et al., 2020; Yu et al., 2016; Zhang et al., 2017; Zhang et al., 2021). For example, the exogenetic sulphuric acid and nitric acid generated by human activities accelerate the dissolution of carbonate rocks in the rivers (Han & Liu, 2004; Li & Zhang, 2008; Li et al., 2010, 2011b; Ma et al., 2023), and therefore increases the concentrations of Ca^2+^, Mg^2+^, and HCO_3_^−^; The average Cl^−^/Na^+^ molar ratio of rivers is higher with more urbanized (Tong et al., 2018), and The Cl^–^, Na^+^ and K^+^ are relatively high in rivers that significantly affected by municipal sewage discharge (Qin et al., 2018); The application of agricultural fertilizer will significantly increase the K^+^, SO_4_^2–^, NO_3_^–^, and Cl^–^ concentrations in the river water (Etchanchu & Probst, 1988), and the SO_4_^2–^, Cl^–^, and Na^+^ contents are relatively high in rivers on which industrial and mining wastewater have impacts (Zhang et al., 2017). The intensification of human activities has led to a significant increase in the content of major ions in rivers, which has gradually become the dominant factor(Chen et al., 2020; Tian et al., 2019; Xu & Liu, 2007; Zeng et al., 2022; Zheng et al., 2022). Moreover, previous studies have shown that agricultural land and coal mines, municipal sewage, *etc*. may be natural sources of release of trace elements in the water environment (Li et al., 2018; Njuguna et al., 2020; Wang et al., 2017). High concentrations of trace elements in the aquatic environment pose potential risks (Zhan, Wu & Jin, 2022). Long-term contact with a high concentration of nitrate-nitrogen may increases the risk of diseases and health effects such as methemoglobinemia, diabetes, thyroid disease, and gastric cancer (Danni et al., 2019; Hord, 2011; Sanchez, Szynkiewicz & Faiia, 2017). Excessive exposure to high fluoride concentrations of fluoride in drinking water can lead to dental fluorosis and skeletal fluorosis (Dehbandi, Moore & Keshavarzi, 2018; Li et al., 2019; Wang et al., 2020). Therefore, polluted rivers have become an environmental hazard and possibly pose a threat to human health (Singh, Malik & Sinha, 2005; Vadde et al., 2018; Wang et al., 2007). It is urgent to carry out risk assessment of rivers, and the most commonly used method is health risk assessment model and evaluation standard provided by the United States Environmental Protection Agency (USEPA) (Gao et al., 2021).

However, it is difficult to elaborate the influence of individual factors on the hydrochemistry and water environmental risk of rivers in depth as the influencing factors of hydrochemistry and water environment risk in the river water are complicated and interrelated (Tong et al., 2018). Existing studies indicate that the small stream is the preferred site to deeply study of the main ion composition sources and risk assessment of river water due to its small size and rapidly responding to external disturbances (Benstead & Leigh, 2012; Crawford et al., 2015). Therefore, we perform data from the (Youyu stream and the Jinzhong stream) (main inflow tributaries of Aha Lake) to investigate the influences of different types of anthropogenic activity on the river hydrochemistry and water environment risk. The main aims are to: (1) discuss the hydrochemical characteristics of small watersheds (2) explore the main ion sources and ratio characteristics of streams, and (3) evaluate potential health risks under the influence of different anthropogenic activities.

## Materials and Methods

### Study area

**Figure 1:**
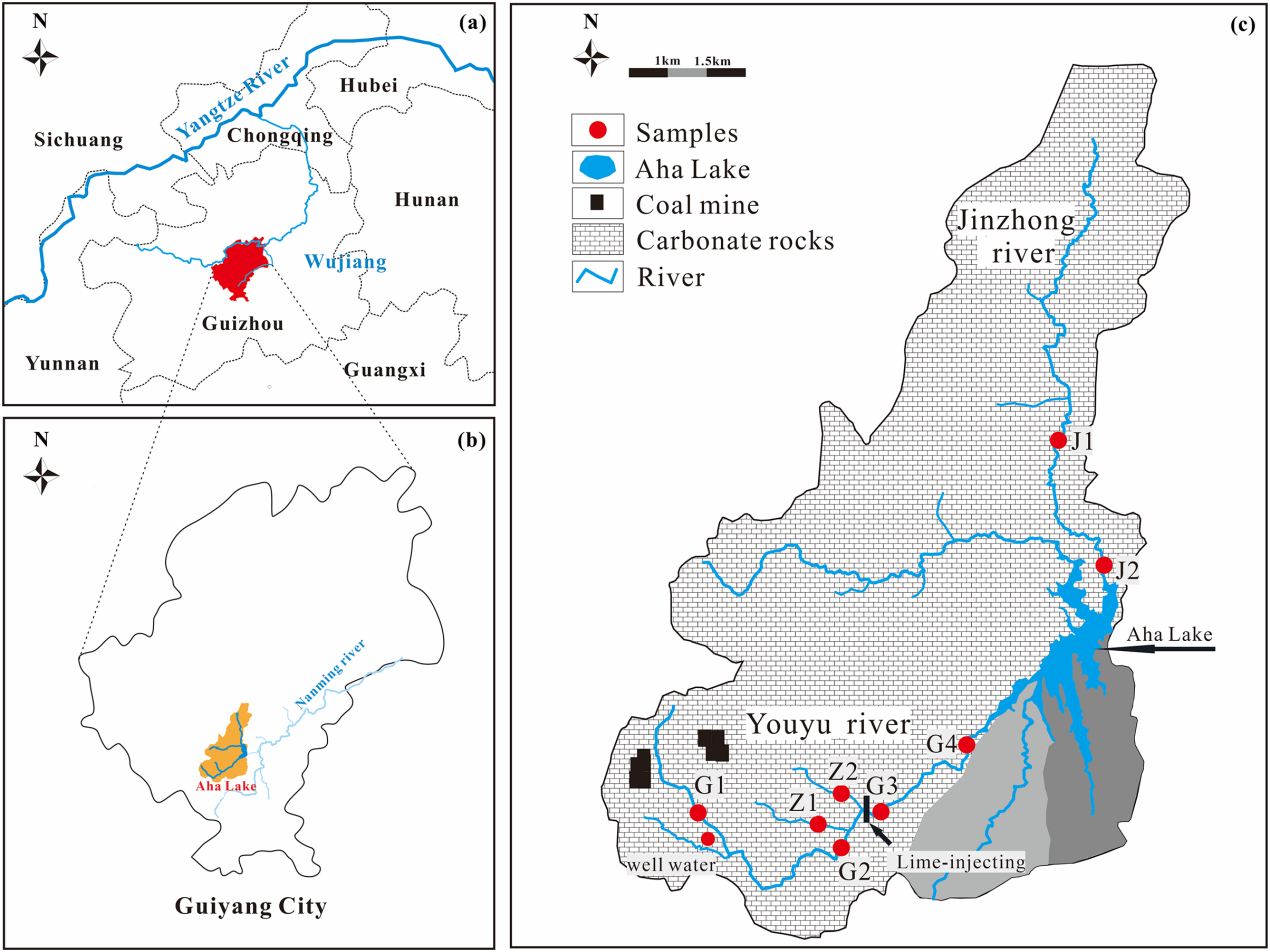
Location of Aha Lake in Southwest China (A and B) and map of the streams and sampling locations (C).

The Youyu stream and the Jinzhong stream are located at Guiyang city (the capital of Guizhou Province, China) and the main tributaries of Aha Lake (drinking water sources of Guiyang City). They join the Nanming river in Wujiang water system which is a tributary of the Yangtze River in the southwest China (Fig. 1). The region of the two streams has typical Karst topography in which primary weathering rock is carbonate minerals as the bedrock is mainly dolomite and limestone. They are significantly affected by anthropogenic activities. Particularly, agricultural land and abandoned coal mines have significantly impact on Youyu stream; however, Jinzhong stream (a typical urban stream) is influenced by municipal wastewater. These two streams have different hydrochemical characteristics for affected by different types of anthropogenic activity. The climate is subtropical monsoon with annual mean precipitation of 1,140–1,200 mm and annual mean air temperatures of 15.3 °C. The Youyu stream flows for a length of 18.5 km, and the catchment area is 65.9 km^2^. The cultivated land and forest land are the main land use types in the basin, which account for 51.74% and 36.10%, respectively. By comparison, population, industrial, mining field, transportation land and gardens, meadows and other land use types account for 3.29%, 0.21% and 8.66%, respectively. The farmland around the Youyu stream is mostly dominated by flat and gently sloping cultivated land, especially the tributary where Z2 is located. Due to the application of agricultural chemicals and extensive production activities, the agricultural area-source pollution is relatively worse than other lands. The upper reach of the Youyu stream once is a dense area of coal mines of Guiyang City. Fe, Mn, pH, and chromaticity values in the rivers seriously exceed their standards because wastewater is perennially discharged from the pits of abandoned coal mines, which indicate the river water is obviously affected by the wastewater of abandoned mines.

The Jinzhong stream flows for a length of 18.5 km, and the catchment area is 5.9 km^2^, which is the tributary with largest discharge of domestic wastewater and the most serious pollution in the Aha Lake basin. The main land use type along the Jinzhong stream are town, village, and industrial land respectively, which accounts for 66.37% of the land area in the watershed; followed by forestland, transportation land, and cultivated land, which, respectively, which account for 11.02%, 9.08%, and 8.45% of land area in the watershed. Gardens, meadows, and other land use types account for 5.08% of the land area in the watershed. The region that the Jinzhong stream flows through is the main urban area of Guiyang City, and the urbanization degree in the watershed is relatively high. It is a typical urban river.

### Sampling and measurements

Sampling campaign implemented occurred during the flood season of rivers in June-August of 2017, with a total of 23 samples (eight samples month in July and August, and except for the G2 point in June). The sampling sites are shown in Fig. 1. Specifically, 17 samples were collected from the Youyu stream, and six samples were collected from the Jinzhong stream. Water samples were collected at 0–20 cm below the surface using washed high-density polyethylene bottles and filtered (0.45 μm nitrocellulose filter) in the field. Temperature (T) and pH were measured *in situ* using WTW340i multi-parameter water quality meters (made in Germany). The HCO_3_^–^ was determined by titration with HCl (0.025 mol•L^–1^) on the day of sampling, and the error was within 5%. Samples were stored and sealed in a 4 °C refrigerator for storage before measurement. Major ions (Na^+^, K^+^, Ca^2+^, Mg^2+^, Cl^−^, NO_3_^−^, F^−^, and SO_4_^2–^) were analyzed and determined by ion chromatograph (IC) (DIONEX, ICS-1100, IonPac AG-19 anion exchange cartridge, IonPac CS-12A cation exchange cartridge), and SiO_2_ was measured using the molybdate yellow photometric method. The analytical precision was within ±5%.

For δ^34^S of sulfate analysis, water was first acidified to pH<2 using distilled 6M HCl. 10% BaCl_2_ solution were added to water samples to convert the dissolved SO_4_^2−^ into BaSO_4_ precipitation, and BaSO_4_ solids were obtained after the samples were filtered through 0.22 μm quantitative filter paper using ultrapure water (Milli-Q, 18.2 MΩ.cm) to flush the residues until the filtrates did not contain Cl^−^ (as determined using the AgNO_3_ test). Then, the BaSO_4_ precipitation on membranes was further calcined (800 °C, 40 min) to gain the BaSO_4_ solid. The δ^34^SSO_4_ composition of BaSO_4_ was determined by analysing SO_2_ gas using a continuous flow isotope ratio mass spectrometer (DELTA V Advantage; Thermo Fisher, Waltham, MA, USA) with an accuracy better than 0.3‰ was performed in the laboratory of College of Resources and Environmental Engineering, Guizhou University.

### Health risk assessment (HRA)

Water pollutants mainly through drinking water and skin contact into the human body, damage to human organs, threatening human health (Xia et al., 2021). As non-carcinogenic pollutants, NO_3_^–^ and F^–^ are often used to assess non-carcinogenic health risks in river waters. The hazard quotient (HQ) is usually used to evaluate noncarcinogenic health risks, and its calculation methods refer to Eqs. (1) and (2).

(1) $${\rm {HQ = ADD/R}}f\rm{D}$$

(2) $${{\rm{ADD_{ingestion}}= C \times IR \times EF \times ED/(BW \times AT)}}$$

where the ADD_ingestion_ is the ingestion intake of daily doses, R*f*D is the average reference dose of daily exposure doses, the reference doses of NO_3_^−^ and F^−^ are reported as 1.6 and 0.04 ppm/day (Li et al., 2016b), respectively, C is the concentrations of ions (mg/L), IR is the rate of daily ingestion (0.7 L/day for children, 1.5 L/day for adults), EF is the exposure frequency (days/year), ED is the exposure duration (12 to 25 years people), BW is the body weight (16 kg for children and 56 kg for adults) and AT is the average time (4,380 and 10,950 days for children and adults) (Wu & Sun, 2016). The residents are threatened by non-carcinogenic contaminants while the values of HQ>1.

### Data analysis

The histogram and triangle diagram were applied to investigate the relationship among the concentrations of ions and the physicochemical parameters in the Youyu stream and Jinzhong stream by the Statistics software package of SPSS 23.0 (IBM SPSS Statistics, Chicago, IL, USA), and Origin 2022 (OriginLab, Northampton, UK) was used for editing graphics.

## Results

### Major ions composition of streams

Chemical compositions were presented in Table 1. The water average temperature of the Youyu stream was 20.98 °C, and the pH ranged from 6.65 to 8.39 (averaged 7.82). The water average temperature of the Jinzhong stream was 20.97 °C; the pH ranged from 7.94 to 8.37 (averaged 8.17). The water in the two rivers were mildly alkaline, revealed that the riverine total alkalinity was mainly composed of bicarbonate alkalinity. There were great differences of TDS (Total Dissolved Solids) in these two streams. The TDS of the Youyu stream ranges from 387.25–1,573.17 mg·L^−1^, averaged 746.80 mg·L^−1^; the TDS of the Jinzhong stream ranges from 428.38–559.77 mg·L^−1^, with an average of 485.78 mg·L^−1^. Compared to other rivers that are also located in the karst region, the TDS values of the two streams are higher than the other rivers (Chetelat et al., 2008; Karim & Veizer, 2000; Li, Lu & Bush, 2014; Li et al., 2011a; Lü et al., 2018; Wu et al., 2008; Zhang et al., 2007). In particular, the TDS of the Youyu stream is comparable to that of the sampling site in the Qingshuijiang River basin in Guizhou province, which is obviously polluted by phosphate mines (740.80 mg·L^−1^) (Lü et al., 2018), and it is higher than that of the Rhine River, which is significantly influenced by industrial activities and mines (47.4–580 mg·L^−1^) (Buhl et al., 1991), ultimately exhibiting the characteristics of a polluted water body.

**Table 1:** Major ionic compositions of Youyu stream and Jinzhong stream.

Sampling site	Eigenvalue	pH	*t*	EC	TDS	Ca^2+^	Mg^2+^	Na^+^	K^+^	HCO_3_^−^	SO_4_^2–^	Cl^–^	NO_3_^–^	F^–^	δ^34^S
			°C	µs/cm	mg•L^–1^	mmol•L^–1^									‰
Youyu stream	Min	6.65	17.60	1,759.00	387.25	1.94	0.27	0.11	0.04	0.54	0.91	0.03	0.03	0.01	−5.68
	Max	8.39	25.40	254.00	1,573.17	8.41	1.94	0.40	0.19	3.14	11.64	0.21	0.31	0.02	−8.23
	Mean	7.82	20.98	847.53	746.80	4.23	0.87	0.23	0.09	2.12	4.13	0.11	0.14	0.01	−7.44
	Std	0.49	2.27	433.74	384.00	2.10	0.59	0.09	0.03	0.75	3.28	0.05	0.08	0.00	1.09
Jinzhong stream	Min	7.94	17.80	526.00	428.38	1.73	0.60	0.51	0.16	3.26	0.83	0.38	0.12	0.02	0.38
	Max	8.37	25.00	677.00	559.77	2.26	0.94	0.80	0.29	4.67	1.13	0.53	0.41	0.03	1.05
	Mean	8.17	20.97	589.00	485.78	1.94	0.76	0.68	0.24	3.81	0.97	0.43	0.23	0.03	0.71
	Std	0.18	2.59	61.13	57.96	0.19	0.12	0.10	0.04	0.55	0.13	0.06	0.12	0.00	0.47

**Figure 2:**
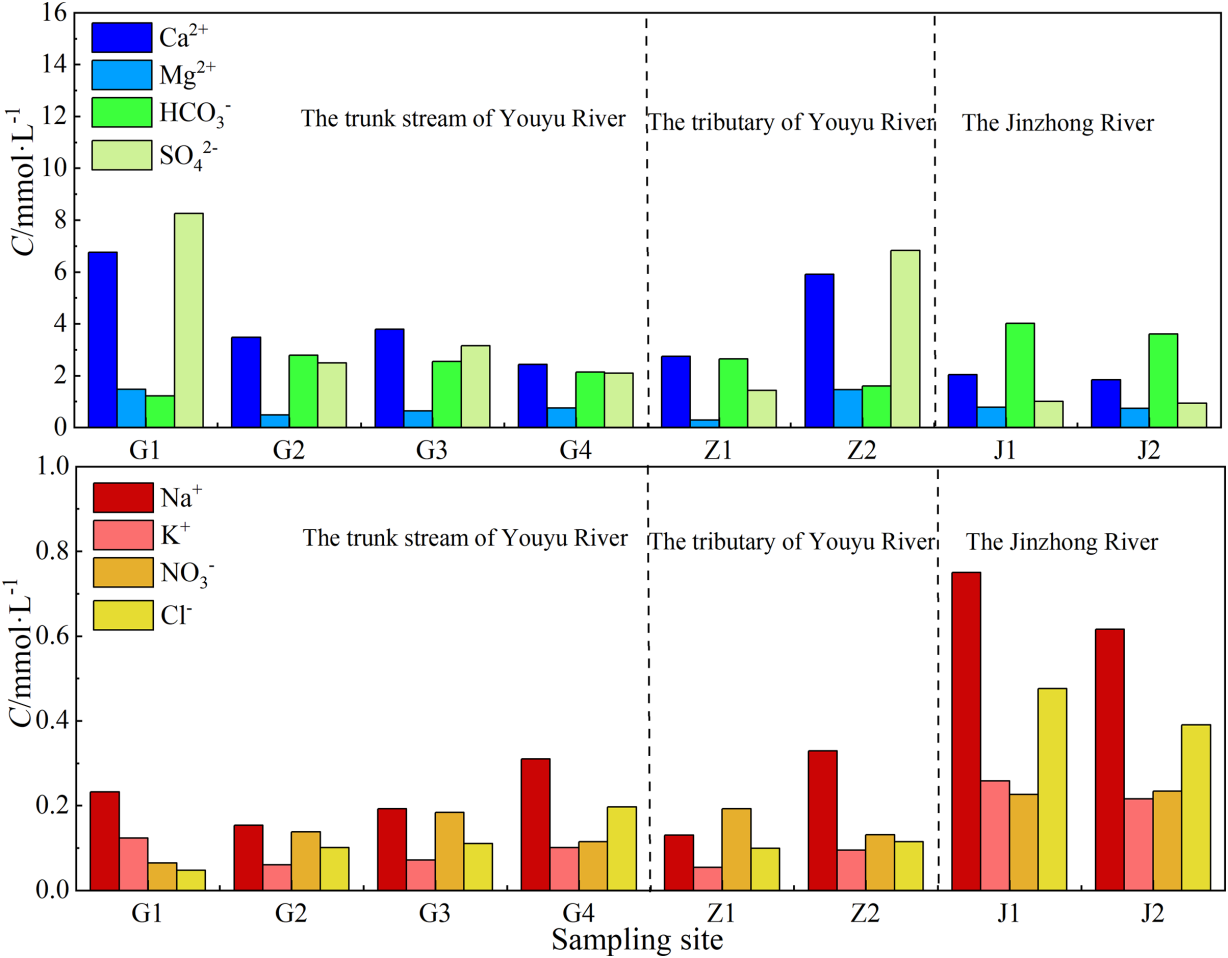
Variation in ion concentrations of Youyu stream and Jinzhong stream.

The trend of main cations in the two streams is the same (both are Ca^2+^>Mg^2+^>Na^+^>K^+^) (Fig. 2), but different in main anions (Youyu stream: SO_4_^2–^>HCO_3_^–^>NO_3_^–^>Cl^–^>F^–^; Jinzhong stream: HCO_3_^–^>SO_4_^2–^>Cl^–^>NO_3_^–^>F^–^). The main cations Ca^2+^, Mg^2+^ account for 78.04%, 16.05% (Youyu stream) and 61.42%, 24.03% (Jinzhong stream) of total cations, respectively. However, the main anion in the Youyu stream is SO_4_^2–^, which accounts for 73.66% of total anions, and HCO_3_^–^ only accounts for 23.96%; while the main anion in the Jinzhong stream is HCO_3_^–^, which accounts for 59.20% of total anions, and SO_4_^2-^ accounts for 30.08%. Moreover, the Youyu stream shows NO_3_^–^>Cl^–^, whereas the Jinzhong stream exhibits Cl^−^>NO_3_^–^. The compositions of Ca^2+^, Na^+^, K^+^, Cl^–^, HCO_3_^–^, and SO_4_^2–^ of the waters in the two stream is obviously different. Ca^2+^ and SO_4_^2–^ in the Youyu stream are significantly higher than those in the Jinzhong stream, but Na^+^, K^+^, Cl^–^, and HCO_3_^−^ in the Jinzhong stream are significantly higher than those in the Youyu stream (Fig. 2).

### Hydrochemical types of streams

**Figure 3:**
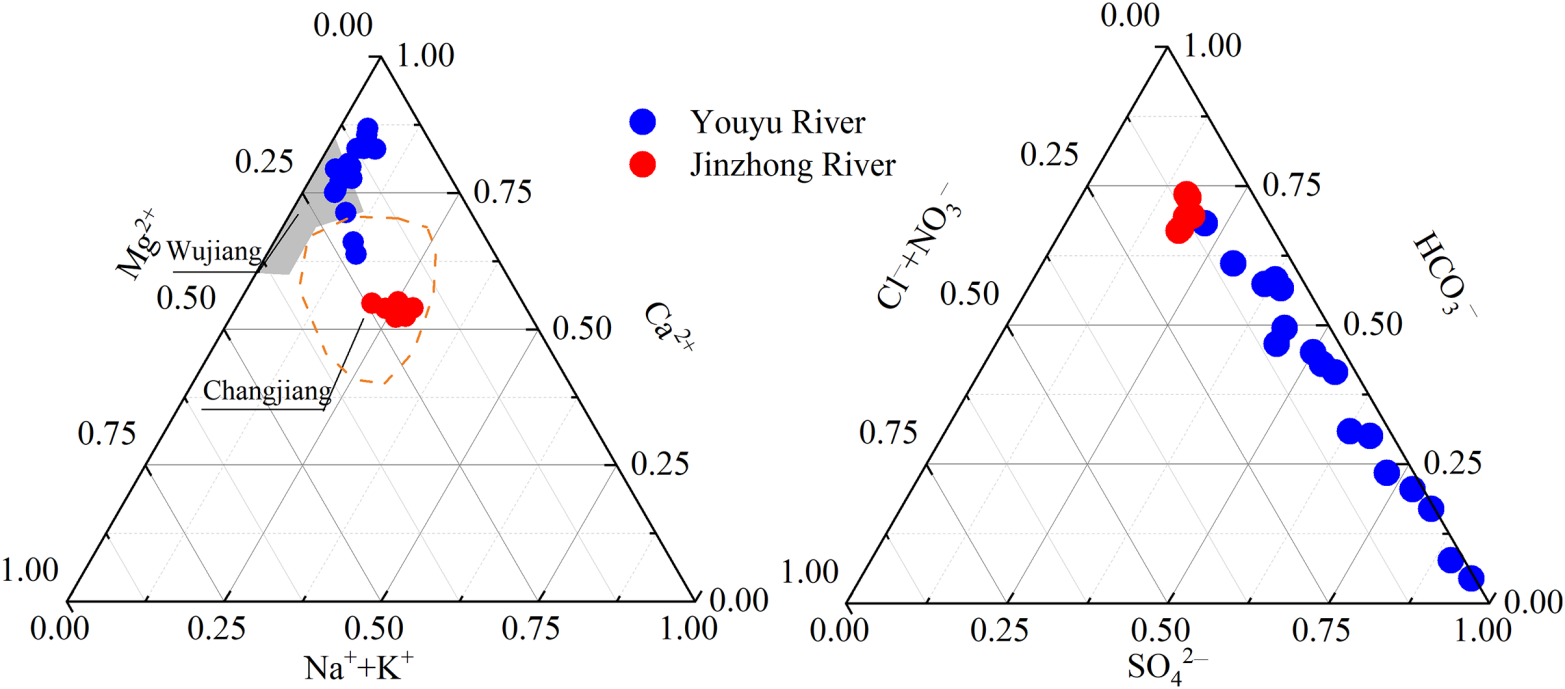
Ternary plots showing the relative abundances of cations and anions (unit: mmol·L^−1^). Data sources are Han & Liu (2004) for Wujiang, and Chetelat et al. (2008) for Changjiang.

As shown in Fig. 3, the sampling sites of river water for the two streams are distributed in different regions, illustrating different hydrochemical characteristics. In the ternary cation plot, sample clusters of the Youyu stream were close to Ca^2+^, which is similar to the Wujiang River (Han & Liu, 2004) and these in the Jinzhong stream were inclined toward the Ca^2+^-Mg^2+^ apex, gradually close to Ca^2+^, which is similar to the Yangtze River Basin of China (Chetelat et al., 2008), belongs to Ca-Mg-HCO_3_ type. Samples the Youyu stream were obviously close to the SO_4_^2−^ in the ternary anion plot, while the anions of the Jinzhong stream are obviously inclined toward HCO_3_^−^, belongs to Ca-SO_4_-HCO_3_ type.

## Discussion

### Composition characteristics and source analysis of Ca^2+^, Mg^2+^, HCO_3_^−^, and SO_4_^2−^ of streams Identification of SO_4_^2−^ sources

**Figure 4:**
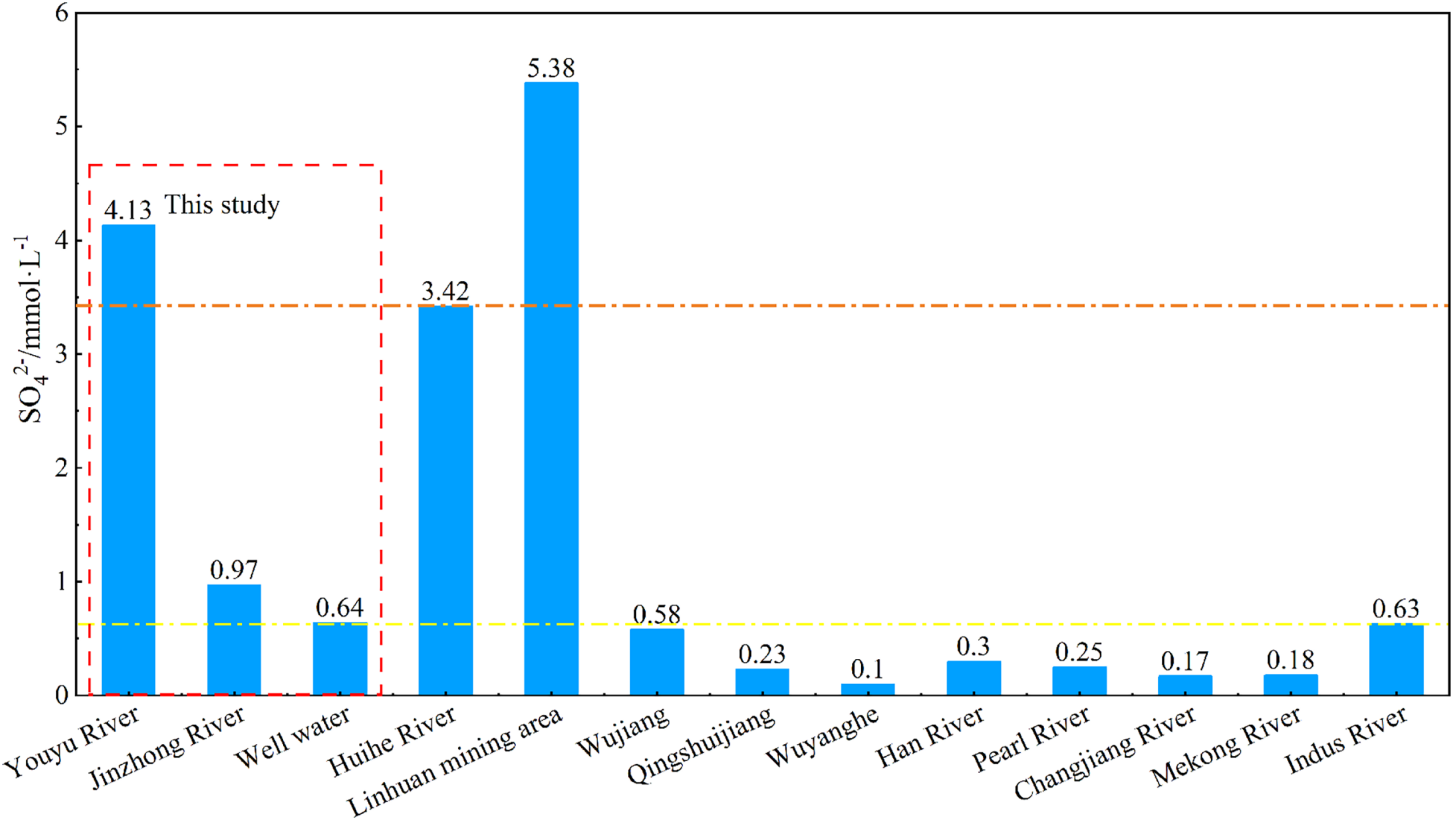
SO_4_^2−^ concentrations of rivers and other rivers across the world. Data sources are Zheng et al. (2019) for Huihe River, Chen et al. (2020) for the Linhuan mining area, Han & Liu (2004) for Wujiang, Qingshuijiang River and Wuyang River, Li, Lu & Bush (2014) for Mekong River, Karim & Veizer (2000) for Indus River, Stallard & Edmond (1983) for Amazon River, Li et al. (2009) for Han River, Zhang et al. (2007) for Pearl River and Chen et al. (2002) for the Yangtze River.

As shown in Fig. 4, the Youyu stream is rich in SO_4_^2−^ (4.13 mmol·L^−1^), which is obviously higher than the Jinzhong stream (SO_4_^2−^: 0.97 mmol·L^−1^) and other rivers in the world (Han & Liu, 2000, 2004; Karim & Veizer, 2000; Li, Lu & Bush, 2014; Li et al., 2009; Stallard & Edmond, 1983; Zhang et al., 2007). Due to the influence of abandoned mine wastewater and Permian coal seams, its SO_4_^2−^ value is between Huihe River (Zheng et al., 2019) and surface water in Linhuan mining area (Chen et al., 2020) polluted by coal mine. And it is important to clarify the source of SO_4_^2−^ under the influence of anthropogenic activities. The SO_4_^2−^ in river waters often has the following main sources: dissolution of evaporite (such as gypsum), oxidation of sulfide, atmospheric input, and industrial activities (Han & Liu, 2005; Liu & Han, 2020; Relph et al., 2021). There were no evaporite sources because the main type of rocks distributed in the region are carbonate rocks. And there is no obvious supply of underground water in the research area, but the SO_4_^2−^ concentration of the Youyu stream is 6.45 times than that of the well water, which demonstrates that the SO_4_^2−^ of this stream is not all from underground water and the oxidation of sulfide. The impact of underground water is not prominent. As shown in Fig. 2, the SO_4_^2−^ has the highest value in sampling site G1 (where there is a high density of abandoned mines but few industrial activities); and it is decreased significantly from upstream to downstream, which reveals that the large amount of SO_4_^2−^ in the river water mainly comes from abandoned mine wastewater discharge (Zheng et al., 2019). During the high-water period, the large amount of mine wastewater flows out with the rainfall increases and increases the SO_4_^2−^ concentration in river water. The SO_4_^2–^ concentration of the Jinzhong stream is obviously lower than that in the Youyu stream and slightly higher than other rivers in the world (Han & Liu, 2000, 2004; Karim & Veizer, 2000; Li, Lu & Bush, 2014; Li et al., 2009; Stallard & Edmond, 1983; Zhang et al., 2007). The Jinzhong stream flow through the main urban area of Guiyang City; due to urbanization, there is abundant construction in progress throughout the watershed, interpreted that in addition to atmospheric and sulfide oxidation sources. Other SO_4_^2−^ derived from urban industrial activity.

**Figure 5:**
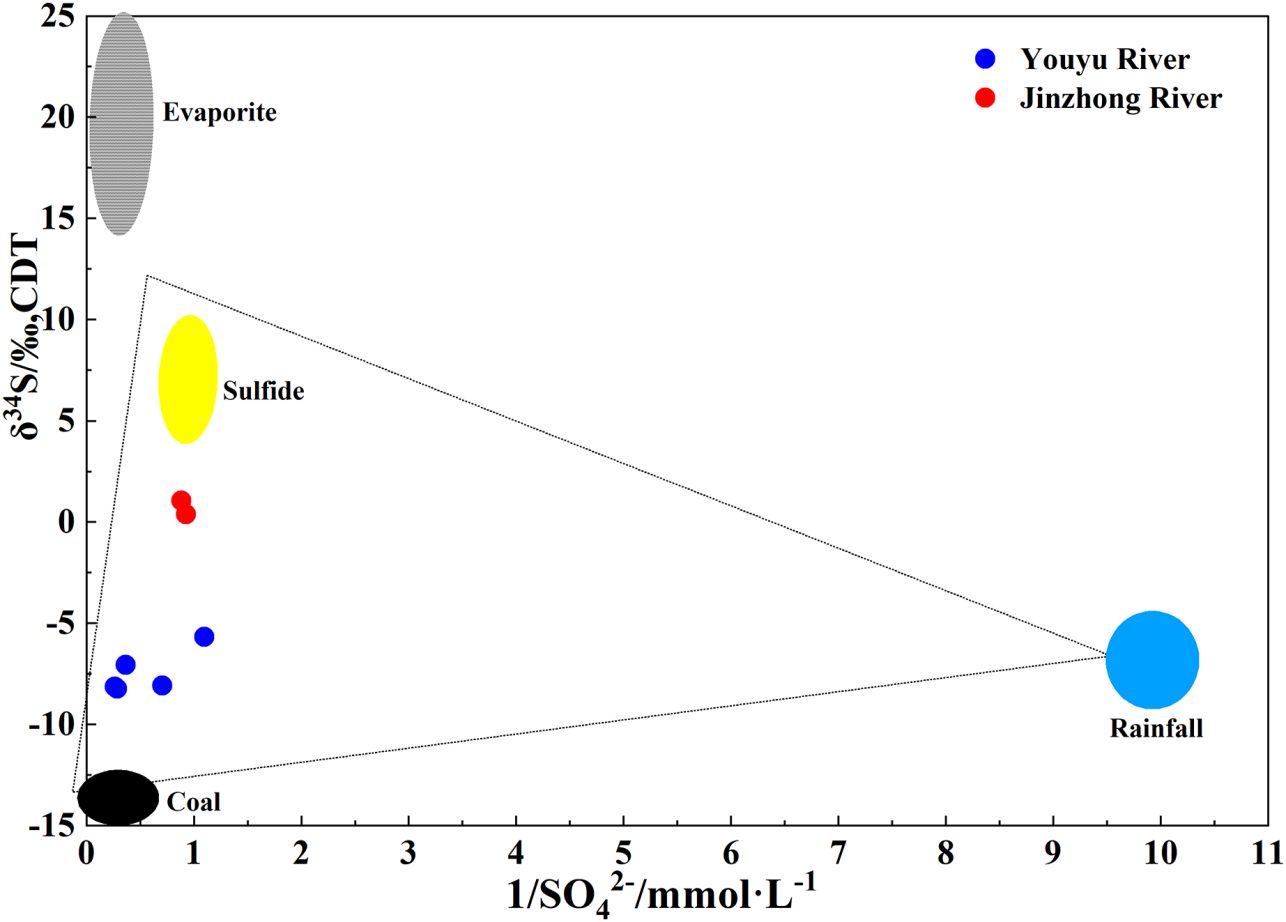
Variations in sulphur isotopic compositions with the reciprocal of the SO_4_^2−^ concentration in Youyu and Jinzhong streams.

Previous studies have shown that the chemical composition of areas far from the ocean is basically unaffected by marine input (Stallard & Edmond, 1981), and the SO_4_^2**–**^ input from inland areas through precipitation is mainly derived from local human activities. In order to analyze the source of SO_4_^2**–**^ in river water, we tested and analyzed the δ^34^S isotope of river water in June. As shown in the Fig. 5, the δ^34^S in the of Youyu stream was between −5.68‰ and −8.23‰, with an average of −7.44‰, is mainly distributed near the coal end element and in the range of rivers polluted by Guizhou coal mines (Cao et al., 2018; Li et al., 2018). Which indicates the SO_4_^2**–**^ ions in the river water are mainly affected by the coal mine wastewater. While the Jinzhong stream has a completely positive δ^34^S value (0.38–1.05‰), is mainly distributed near the sulfide end element, and the SO_4_^2**–**^ ions in the river water are mainly affected by sulfides. In general, the isotope study results are the same as the previous analysis of sulfuric acid source.

### Sources of Ca^2+^, Mg^2+^, and HCO_3_^−^ and analysis of characteristics

**Figure 6:**
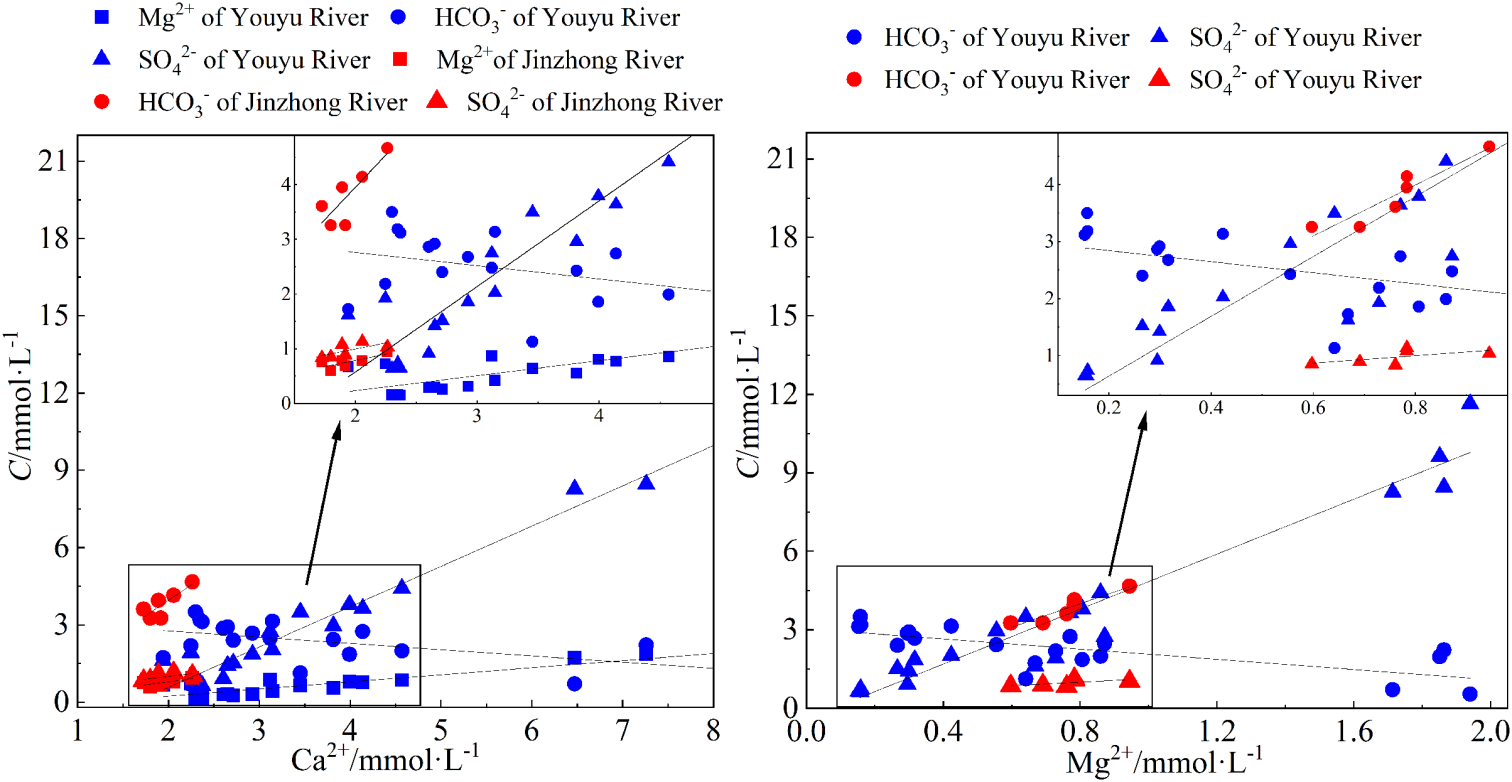
Relationship between Ca^2+^, Mg^2+^ and HCO_3_^−^, SO_4_^2−^ of waters in Youyu and Jinzhong streams.

Ca^2+^, Mg^2+^, and HCO_3_^**–**^ in karst areas are usually derived from the weathering of carbonate rocks, weathered mainly by carbonic acid and sulfuric acid (Lenart-Boroń et al., 2017). Generally, the ratio of 2(Ca^2+^+Mg^2+^)/HCO_3_^−^ is close to one while H_2_CO_3_ weathers the carbonate rocks; the ratio of 2(Ca^2+^+Mg^2+^)/HCO_3_^−^ is close to two and the ratio of 2SO_4_^2**–**^/HCO_3_^**–**^ is close to one while H_2_SO_4_ weathers the carbonate rocks (Li et al., 2011a; Liu et al., 2008). As shown in Fig. 6, the changing trends of Ca^2+^, Mg^2+^, and HCO_3_^**–**^ in the Jinzhong stream are the same, and show positive correlation with each other (*r* = 0.83, *P* < 0.05; *r* = 0.936, *P* < 0.01), but no obvious correlation with SO_4_^2−^, demonstrated the dissolution of carbonate weathering. It is same as the chemical characteristics of karst rivers. The main anion of the Jinzhong stream is HCO_3_^−^, the ratio of 2(Ca^2+^+Mg^2+^)/HCO_3_^**–**^ in the river ranges from 1.40 to 1.44, and the ratio of 2SO_4_^2**–**^/HCO_3_^**–**^ ranges from 0.50-0.51, indicated that the Ca^2+^, Mg^2+^, and HCO_3_^**–**^ were less influenced by sulphuric acid.

In the Youyu stream, Ca^2+^ and Mg^2+^ are positively correlated with SO_4_^2**–**^ (*r* = 0.98, *P* < 0.01; *r* = 0.99, *P* < 0.01), whereas Ca^2+^ and Mg^2+^ are negatively correlated with HCO_3_^**–**^ (*r* = −0.61, *P* < 0.01; *r* = −0.72, *P* < 0.01) (Fig. 6), exhibiting abnormal characteristics of rivers in the karst region. Then, the ratio of 2(Ca^2+^+Mg^2+^)/HCO_3_^**–**^ in the river water ranges from 2.30 to 18.60, and the 2SO_4_^2**–**^/HCO_3_^**–**^ ratio ranges from 1.10 to 19.61, reveals that sulfuric acid apparently participated in the weathering of carbonate rocks in the watershed. The abundant rainfall during the periods of high water causes a large amount of SO_4_^2−^ to flow into the river water, thereby increasing the SO_4_^2−^ concentration in the river water. This makes the equilibrium of chemical reaction (3) proceed to the left, and causes significant CaCO_3_ deposition (Tang, Jin & Liang, 2021). Meanwhile, the acid condition will transform HCO_3_^**–**^ in the river water to H_2_CO_3_ and will reduce the HCO_3_^**–**^ concentration. At the same time, the large amount of H_2_CO_3_ and H_2_SO_4_ in the river water will reinforce the dissolution of carbonate minerals in the watershed and will make the chemical reaction proceed to the right (Niu et al., 2022; Ren et al., 2019). especially in the G1 and Z2 points, the Ca^2+^ and Mg^2+^ in the Youyu stream were significantly higher than those in other sampling points.

(3) $${\rm{C}}{{\rm{a}}_{\rm{x}}}{\rm{M}}{{\rm{g}}_{({\rm{1 - x}})\,}}{\rm{C}}{{\rm{O}}_{\rm{3}}}{\rm{ + }}\,{{\rm{H}}_{\rm{2}}}{\rm{C}}{{\rm{O}}_{\rm{3}}}{\rm{ + }}\,{{\rm{H}}_{\rm{2}}}{\rm{S}}{{\rm{O}}_{\rm{4}}}\,{\rm{ = }}\,\,{\rm{3xC}}{{\rm{a}}^{{\rm{2 + }}}}{\rm{ + }}\,{{\rm{3}}_{({\rm{1 - x}})}}{\rm{M}}{{\rm{g}}^{{\rm{2 + }}}}{\rm{ + }}\,{\rm{4HCO}}{{\rm{3}}^{\rm{ - }}}{\rm{ + }}\,{\rm{SO}}_{\rm{4}}^{{\rm{2 - }}}$$

### Characteristics of NO_3_^–^, Na^+^, K^+^, and Cl^−^ in the river water and source analysis

#### Characteristics of NO_3_^–^ and source analysis

Pollutants associated with anthropogenic activity are rich in K, Ca, S, Cl, and N (Stallard & Edmond, 1981). Because K, Ca, S, and Cl are also produced by rock weathering, NO_3_^**–**^, the existing form of N in waters can be used as the characteristic ion to characterize the influence of anthropogenic activity (Han & Liu, 2000). Generally, NO_3_^**–**^ mainly comes from the application of nitrogen fertilizer in agriculture, industrial activities, and nitrogen and oxygen compounds generated by the vehicle exhaust (Hu, Zhou & Xia, 2011; Zeng et al., 2023). The main land use type of Youyu stream (accounting for 51.74%) is cultivated land, the Jinzhong stream is a typical urban river, and agricultural cultivated land only accounts for 8.45% of land area in the watershed. However, the NO_3_^**–**^ in Jinzhong River (0.23 mmol·L) which is similar to the Wujiang River (Han & Liu, 2004) and these in the Jinzhong stream were inclined toward the Ca^2+^-Mg^2+^ apex is obviously higher than that in Youyu River (0.16 mmol·L^−1^) (Fig. 2), which shows the relatively high concentration of the nitrate radical in the river water likely does not all come from agricultural activities. Urban wastewater discharge is the main source of nitrate nitrogen in urban rivers. The study indicated that wastewater discharge is the main source of nitrogen in urban rivers (Yu et al., 2016) in which urban sewage contains relatively high NO_3_^**–**^ and Na^+^ (Ding et al., 2017). In addition, NO_3_^−^ exhibits an obvious positive correlation with increasing urban construction land (Ahearn et al., 2005; Lenart-Boroń et al., 2017). The Jinzhong stream flows through the main urban area of Guiyang City, where the population density is relatively large and the urbanization degree is relatively high. It is the river with the largest discharge of domestic sewage in the Aha Lake Basin. It is apparent that for NO_3_^**–**^ in the Jinzhong stream, except for a small amount that comes from agricultural activities, the vast majority comes from the discharge of urban sewage.

### Characteristics of Na^+^, K^+^, and Cl^–^ and source analysis

**Figure 7:**
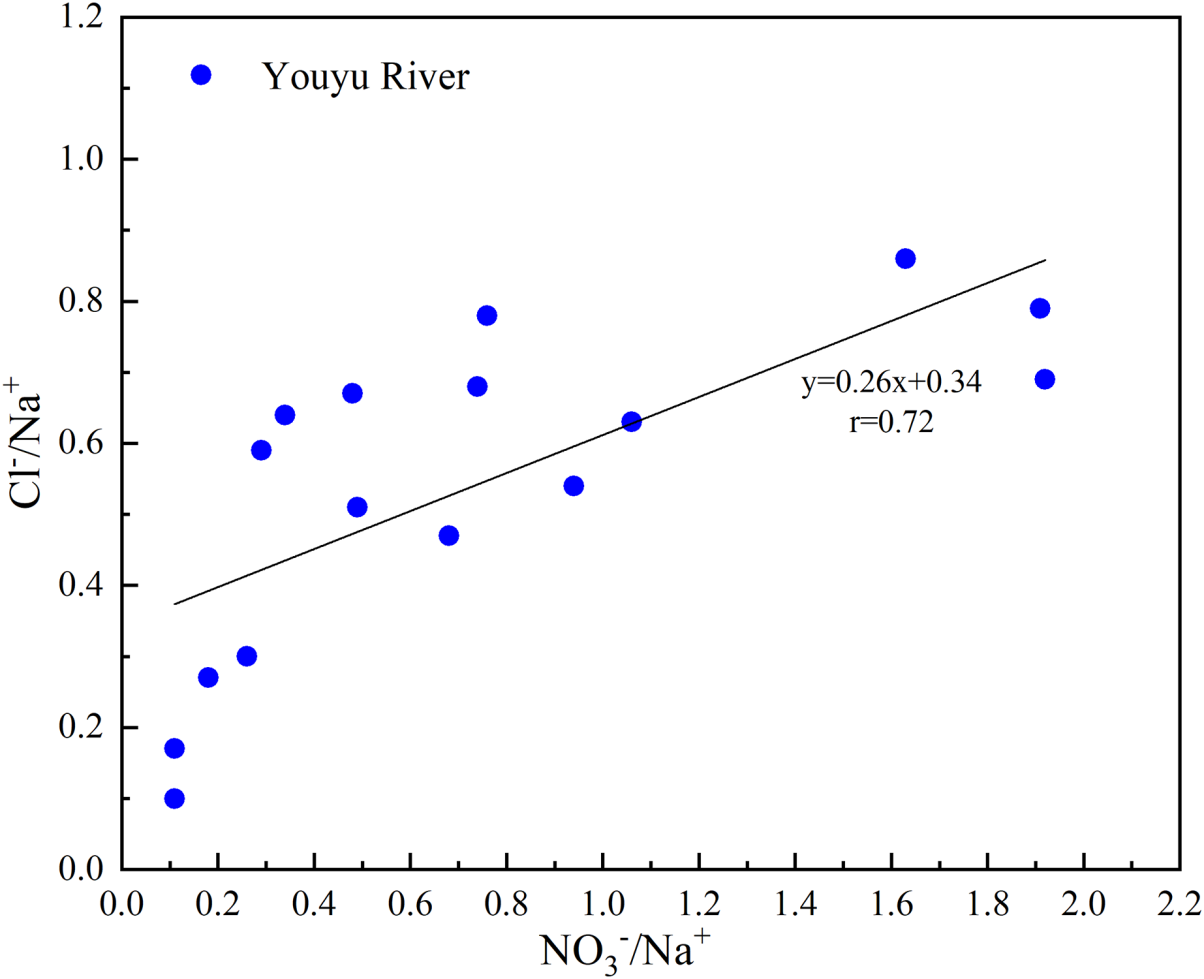
Plots of Cl^–^/Na^+^ *vs*. NO_3_^–^/Na^+^ of rivers that flow into Aha Lake.

For the underground water in the area underlain by carbonate rocks, the Cl^–^ content is very low, and Cl^–^ in natural conditions mainly comes from atmospheric precipitation and anthropogenic activity (Ge et al., 2021; Lang et al., 2005). Urban domestic sewage is rich in Na^+^ and Cl^–^, and K^+^ usually comes from the application of agricultural potassium fertilizer (KCl) (Ge et al., 2021; Grosbois et al., 2000; Liu et al., 2006; Sheikh et al., 2014; Widory et al., 2005). The Cl^–^ concentration in the Youyu stream is relatively low (0.11 mmol·L^−1^), which only accounts for 1.69% of total anions in the watershed. The Cl^–^/Na^+^ and NO_3_^–^/Na^+^ in the river water have an obvious linear relationship (Fig. 7), which indicates that Cl^**–**^ and NO_3_^**–**^ have the same source and are also mainly affected by agricultural activities. The Na^+^ and K^+^ concentrations in the Youyu stream are relatively low and only account for 2.65% and 0.66%, respectively, of total cations. Na^+^ and K^+^ have the same trend and an obvious positive correlation relationship (*r* = 0.72, *P* < 0.01). There is no obvious positive correlation between Na^+^, K^+^ and NO_3_^**–**^, Cl^**–**^, which indicates that the main source of Na^+^ and K^+^ in the Youyu stream is not an agricultural source, and the small amount of Na^+^ and K^+^ in the river water likely comes from domestic sewage and industrial wastewater (Han & Xu, 2022; Rao et al., 2019).

The Jinzhong stream has relatively high Na^+^, K^+^, and Cl^**–**^ (Na^+^: 0.68 mmol·L^−1^, K^+^: 0.24 mmol·L^–1^, Cl^–^: 0.43 mmol·L^−1^), which is obviously higher than the Youyu stream, even though they are located in the same geological background and climatic environment. Its Na^+^, K^+^, and Cl^−^ are close to urban rivers (Chetelat et al., 2008) and the urban river site in the Godāvari River during the rainy season (Jha et al., 2009), exhibiting the characteristics of urban rivers. Na^+^, K^+^, and HCO_3_^−^ in the river water of the basin do not have an obvious correlation. This indicates that rock weathering is not the main source of Na^+^ and K^+^ in the river water, and it is possible that the main source of Na^+^ and K^+^ is anthropogenic activity. The Na^+^, K^+^, and Cl^−^ in surface water exhibit a significant positive correlation with residential construction land and cultivated land, as well as a significant negative correlation with forestland (Lenart-Boroń et al., 2017). The Jinzhong stream is a typical urban river, and the Cl^–^/Na^+^ of river water ranges from 0.57–0.74, and the SO_4_^2–^/Na^+^ ranges from 1.33–1.62, which is close to the Cl^–^/Na^+^ (0.66) and SO_4_^2–^/Na^+^ (0.45) ratios (Chetelat et al., 2008) of municipal sewage in Wuhan City, indicating that the main source of Na^+^, K^+^, and Cl^–^ in the river water is the discharge of municipal domestic wastewater.

### Ion composition and ratio characteristics of river water under the influence of mining activities/urban sewage

**Figure 8:**
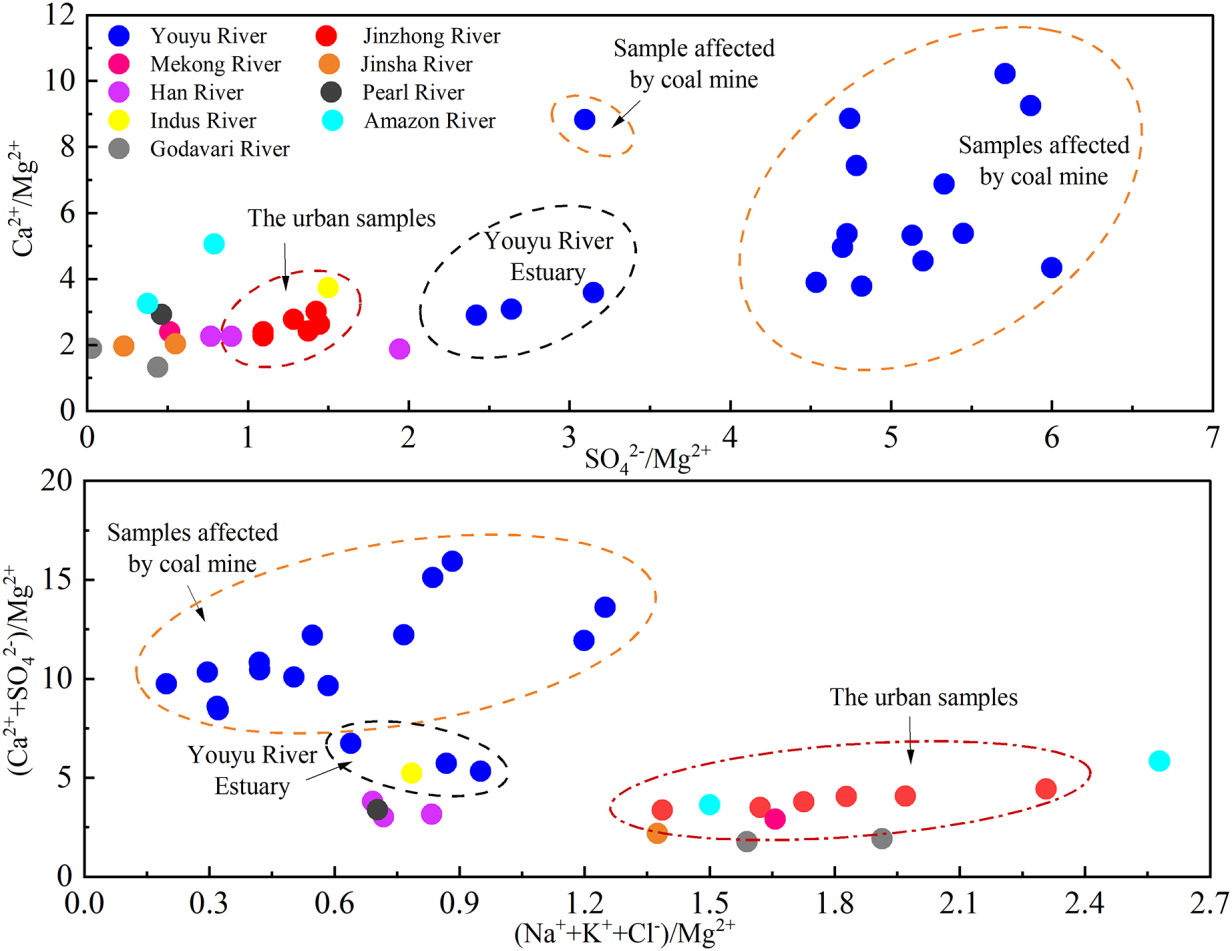
Diagram of the variation in Ca^2+^/Mg^2+^, SO_4_^2−^/Mg^2+^, (Ca^2+^+ SO_4_^2−^)/Mg^2+^, and (Na^+^+K^+^+Cl^−^)/Mg^2+^ ratios. Data sources are Li et al. (2009) and Wu et al. (2008) for Han River, Li, Lu & Bush (2014) for Mekong River, Chetelat et al. (2008) for Jinsha River; Zhang et al. (2007) for Pearl River, Karim & Veizer (2000) for Indus River, Stallard & Edmond (1983) for Amazon River, Jha et al. (2009) for Godāvari River.

The Youyu stream is influenced by industrial and mining activities, and the urban Jinzhong stream is affected by the discharge of municipal domestic wastewater, but the Mg^2+^ is produced by rock weathering and is relatively stable in water (Bisht et al., 2022; He et al., 2022). Therefore, we selected Mg^2+^ to compare and analyze the ion composition characteristics of river water. As shown in Fig. 8, the Youyu stream has relatively high Ca^2+^/Mg^2+^, SO_4_^2−^/Mg^2+^, and (Ca^2+^+ SO_4_^2−^)/Mg^2+^ ratios (Ca^2+^/Mg^2+^: 2.90–10.22; SO_4_^2−^/Mg^2+^: 2.42–6.00; (Ca^2+^+ SO_4_^2−^)/Mg^2+^: 5.32–15.93), which are obviously higher than in the Jinzhong stream basin (Ca^2+^/Mg^2+^: 2.27–3.01; SO_4_^2−^/Mg^2+^: 1.10–1.45; (Ca^2+^+ SO_4_^2−^)/Mg^2+^: 3.36–4.44), the Han River (Li et al., 2009), the Mekong River (Li, Lu & Bush, 2014; Wu et al., 2008), the Jinsha River (Chetelat et al., 2008), the Pearl River (Zhang et al., 2007), the Indus River (Karim & Veizer, 2000), the lower reach of the Amazon River (Stallard & Edmond, 1983) and the Godāvari River (Jha et al., 2009). In particular, the SO_4_^2−^/Mg^2+^ ratio, the SO_4_^2−^/Mg^2+^ ratio of Youyu River (4.61) is 3.58 times higher than Jinzhong stream (1.29), and higher than other rivers in the world (1.97–165.78 times, and the average is 18.20 times). Moreover, the ratio of SO_4_^2−^/Mg^2+^ was higher in the sampling sites seriously polluted by coal mine wastewater (Qian et al., 2018; Tang, Jin & Liang, 2021). Therefore, the higher the SO_4_^2−^/Mg^2+^ in the river water, the more significant the influence of acid mine drainage on it.

Compared to the Youyu stream (0.20–1.25), the Jinzhong stream has the a high (Na^+^+K^+^+Cl^−^)/Mg^2+^ ratio (1.39–2.31), which similar to the ratio characteristics of the urban Godāvari River, which is subject to an obvious influence by anthropogenic activity (pre-rainy season and rainy season), and the Mekong River, Jinsha River, and lower reach of Amazon River, which are subject to the obvious influence of anthropogenic activity input (Chetelat et al., 2008; Li, Lu & Bush, 2014; Stallard & Edmond, 1983). An analysis of the ratio characteristics of two rivers and other rivers in the world affected by urban sewage and mine wastewater indicated that SO_4_^2−^/Mg^2+^ ratio can be used to characterize the influence of coal mines on the river water, and (Na^+^+K^+^+Cl^−^)/Mg^2+^ can be used to characterize the influence of urban sewage on the rivers. The rivers subject to the obvious influence of acidic mine wastewater have relatively high SO_4_^2−^/Mg^2+^ ratio, whereas the rivers subject to the obvious influence of urban sewage has a relatively high (Na^+^+K^+^+Cl^−^)/Mg^2+^ ratio.

### Ion composition and ratio characteristics of river water under the influence of agricultural activities/urban residential activities

**Figure 9:**
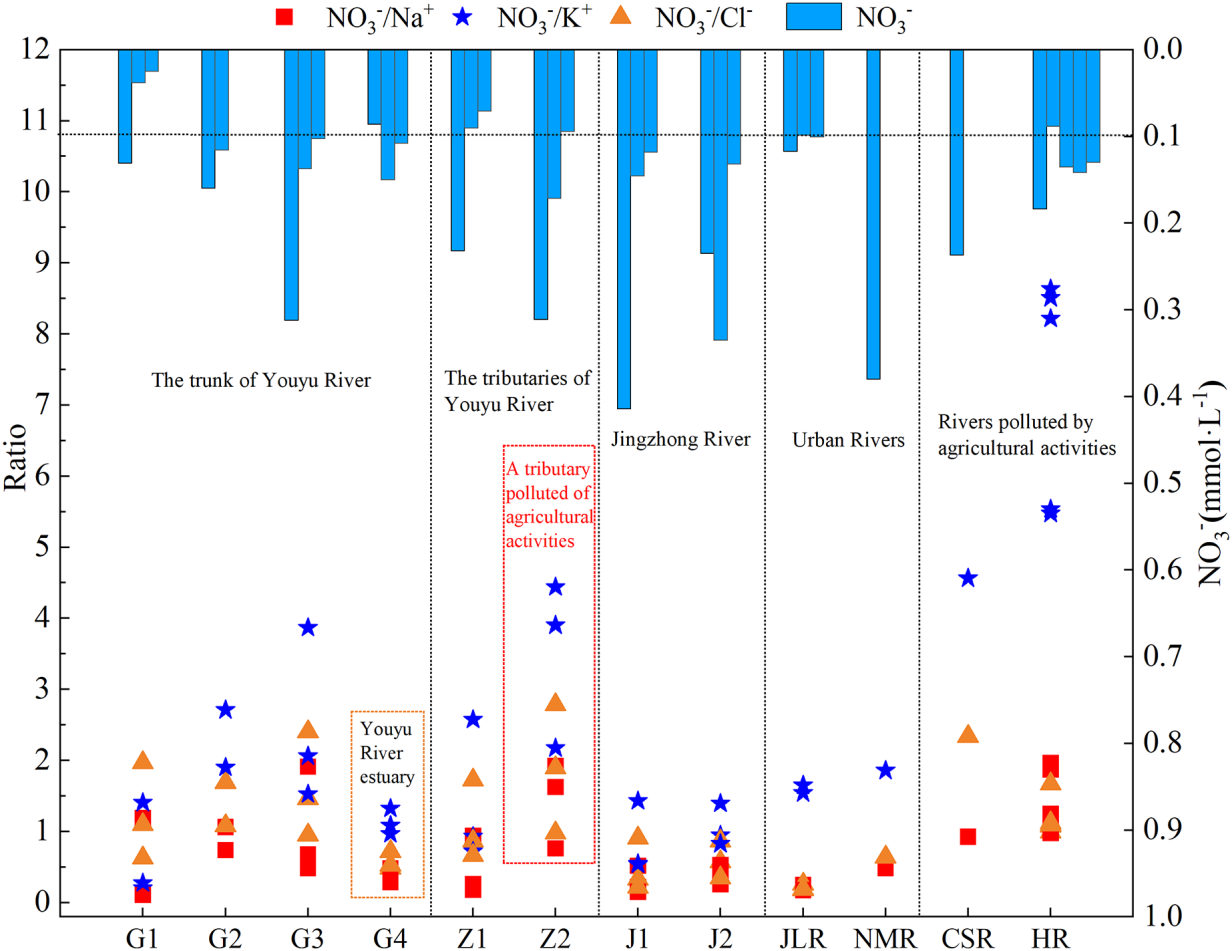
Variation trends of NO_3_^−^/Na^+^, NO_3_^−^/Cl^−^, and NO_3_^−^/K^+^ during the high-water period for the tributaries into Aha Lake. Data sources are Qin et al. (2018) for Jialing River, Han & Xu (2022) for Nanming River (NMR), Ge et al. (2021) for Chishui River (CSR) and Li et al. (2009) for Han River (HR).

The influencing factors and sources for NO_3_^–^, Na^+^, K^+^, and Cl^–^ in the river water of the two tributaries into Aha Lake are different, and have different NO_3_^–^/Na^+^, NO_3_^–^/K^+^, and NO_3_^–^/Cl^–^ characteristics. As shown in Fig. 9, the Youyu stream is obviously influenced by agricultural activities and has relatively high NO_3_^–^/Na^+^, NO_3_^−^/K^+^, and NO_3_^–^/Cl^–^ ratios, which are obviously higher than the urban Jinzhong stream. At point Z2 of the Youyu stream, there is a large amount of dry land, paddy fields, and irrigated land and relatively abundant agricultural land. Due to the relatively strong influence of agricultural activities, the NO_3_^–^/Na^+^, NO_3_^–^/K^+^, and NO_3_^–^/Cl^–^ ratios at point Z2 are 1.44, 3.50, and 1.89, respectively. The characteristics of the ratios are similar to the water sample of rivers studied by Liu et al. (2006) that are subject to the obvious influence of agricultural activities (NO_3_^–^/Cl^–^>1) (Liu et al., 2006), which is also similar to the the Han River and Chishui River that is affected by agricultural activities (Ge et al., 2021; Li et al., 2009) (Fig. 9). The NO_3_^–^/Na^+^, NO_3_^–^/K^+^, and NO_3_^–^/Cl^–^ ratios were higher in the rivers that were greatly affected by agricultural activities.

The NO_3_^–^ content of the Jinzhong stream is higher than that of the Youyu stream (Table 1), the NO_3_^–^/Na^+^, NO_3_^–^/K^+^, and NO_3_^–^/Cl^–^ ratios (NO_3_^–^/Na^+^: 0.33; NO_3_^–^/K^+^: 0.95, NO_3_^–^/Cl^–^: 0.54) are relatively low, and the ratios are obviously lower than those at point Z2 and the Han River and Chishui River, which are subject to the significant influence of agricultural activities (Ge et al., 2021; Li et al., 2009). But it relatively is similar to the urban rivers Jialing River (Tong et al., 2018) and Nanming River (Han & Xu, 2022), and which is also similar to the characteristics of the urban sewage ratio (NO_3_^–^/Na^+^: 0.4) studied by Ding et al. (2017) representing obviously characterize the characteristics of a typical urban river. The analysis of ratios for the two small watersheds and other rivers in the world affected by urban sewage and mine wastewater indicates that the NO_3_^–^/Na^+^, NO_3_^–^/K^+^, and NO_3_^–^/Cl^–^ ratios can be used to differentiate the characteristics of rivers that are influenced by agricultural activities and urban sewage. If the NO_3_^–^ concentration is high, the rivers affected by agricultural activities have relatively higher NO_3_^–^/Na^+^, NO_3_^–^/K^+^, and NO_3_^–^/Cl^–^ ratios compared to urban rivers that are influenced by urban sewage; whereas the rivers with more urban sewage input have relatively low ratios compared to rivers that are subject to the influence of agricultural activities.

### Health risk assessment of tributaries into Aha Lake

**Figure 10:**
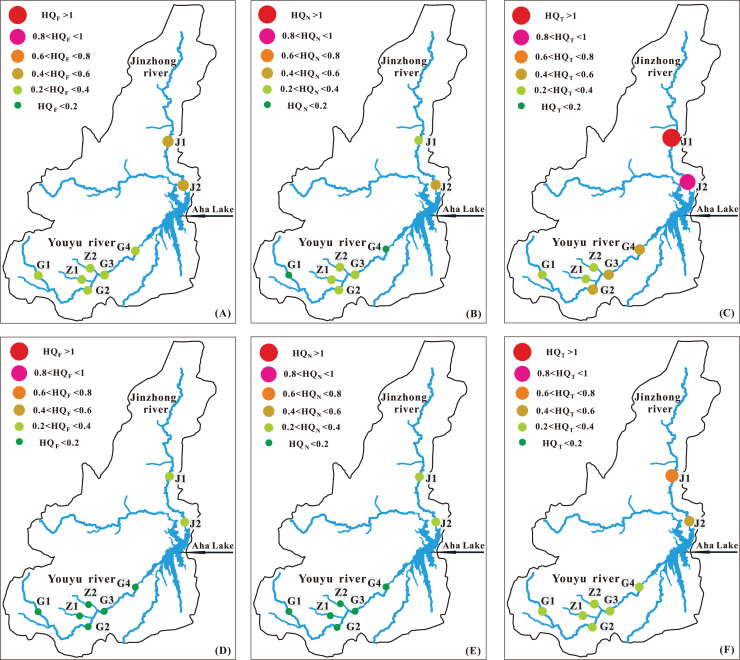
(A) The HQ_F_ for children, (B) the HQ_N_ for children, (C) the HQ_T_ for children, (D) the HQ_F_ for adult, (E) the HQ_N_ for adult and (F) the HQ_T_ for adult in the tributaries into Aha Lake.

Aha Lake is an important drinking water source in Guiyang City, which water quality is mainly affected by its tributaries (Marziali et al., 2021). Therefore, we evaluated the drinking water quality of the two main tributaries (Youyu stream and Jinzhong stream) of Aha Lake. As is shown in Fig. 10, the non-carcinogenic health risks of NO_3_^−^ (HQ_N_) and F^−^ (HQ_F_) in the tributaries into Aha Lake were calculated. The HQ values of children in the Youyu stream exhibited the order of HQ_F_ (mean: 0.24) > HQ_N_ (mean: 0.23) and those of adults exhibited the order of HQ_F_ (mean: 0.15) > HQ_N_ (mean: 0.14). The HQ values of children in the Jinzhong stream exhibited the order of HQ_F_ (mean: 0.58) > HQ_N_ (mean: 0.39) and those of adults exhibited the order of HQ_F_ (mean: 0.35) > HQ_N_ (mean: 0.24). In addition, both HQ_T_ and HQ_N_ for children and adults are higher in Jinzhong stream than in Youyu stream, indicating which is affected by the increase of F^-^ and NO_3_^−^ ion content in river water caused by a large amount of domestic sewage produced by the development of urbanization around Jinzhong stream (Chen et al., 2021; Lockmiller et al., 2019). The HQ values of children were always higher adults than in the Youyu stream and Jinzhong stream, and the total HQ value (HQ_T_) of children was higher than one at J1 in the Jinzhong stream, which shows that children in Jinzhong stream basin are threatened by non-carcinogenic pollutants and Aha Lake is affected by non-carcinogenic pollutants. Previous studies have shown that children may also be potentially harmful when their HQ value is higher than 0.1 (De Miguel et al., 2007). Each HQ value of F^−^ and NO_3_^−^ for children was higher than 0.1 in the tributaries into Aha Lake, indicating that the children may also be potentially endangered. In general, the non-carcinogenic health risk of riverine ions for residents in the Aha Lake is very noteworthy.

## Conclusions

The present study evaluated major ion compositions, ratios, sources and risk assessment of karst stream under the influence of anthropogenic activities. The main conclusions of the present study were as follows: The Youyu stream has relatively high Ca^2+^ (78.04%), SO_4_^2−^ (73.66%) and HCO_3_^–^ (23.96%), belongs to Ca-SO_4_-HCO_3_ type; the Jinzhong stream has relatively high Ca^2+^ (61.42%), Mg^2+^ (24.03%) and HCO_3_^–^ (59.20%), belongs to Ca-Mg-HCO_3_ type. The Youyu stream is subject to an obvious influence by mines has relatively high SO_4_^2–^; the Jinzhong stream is subject to an obvious influence by urban sewage and has relatively high Na^+^, K^+^, and Cl^–^. The NO_3_^**–**^ in the Youyu stream is mainly affected by agricultural activities, whereas NO_3_^**–**^ in the Jinzhong stream is mainly affected by urban sewage. The results of ion ratio show that the ratio of SO_4_^2**–**^/Mg^2+^ in Youyu stream (4.61) polluted by coal mine is much higher than that in Jinzhong stream (1.29), and the ratio of (Na^+^+K^+^+Cl^**–**^)/Mg^2+^ in Jinzhong stream (1.81) polluted by urban sewage is higher than Youyu stream (0.64); moreover, the ratios of NO_3_^−^/Na^+^, NO_3_^−^/K^+^, and NO_3_^−^/Cl^−^ in the agriculturally polluted Youyu stream were higher than those in the Jinzhong stream. We can identify the impact of human activities on rivers by ion ratios (SO_4_^2**–**^/Mg^2+^, (Na^+^+K^+^+Cl^**–**^)/Mg^2+^, NO_3_^−^/Na^+^, NO_3_^−^/K^+^, and NO_3_^−^/Cl^−^). The health risk assessment shows the HQ_T_ and HQ_N_ for children and adults are higher in Jinzhong stream than in Youyu stream, the total HQ value (HQ_T_) of children was higher than one at J1 in the Jinzhong stream, which shows that children in Jinzhong stream basin are threatened by non-carcinogenic pollutants. In the later period, the water environment management of the tributaries of Aha Lake should be strengthened.

## Supplemental Information

10.7717/peerj.15368/supp-1 Supplemental Information 1 Raw data of measured major ion concentration, locations and date of sampling. DO,EC, T, Na^+^, K^+^, Mg^2+^,Ca^2+^, F^−^, Cl^-^, NO_3_^−^, SO_4_^2−^, HCO_3_^−^ and δ^34^S of water in Youyu and Jinzhong streams.

## Additional Information and Declarations

### Data Availability

The following information was supplied regarding data availability: The raw measurements are available in the Supplemental File.
